# 1891. Repeated Assessment of SARS-CoV-2 Seroprevalence among Health Care Workers Early in the Pandemic: Relationship to Workplace Exposures

**DOI:** 10.1093/ofid/ofac492.1518

**Published:** 2022-12-15

**Authors:** Sherry Demian, Dominic O Awuah, Danielle Osterholzer, Lina Altameemi, Carlos Rios-Bedoya, Ahmad Taftaf, Hoang C Nguyen

**Affiliations:** Hurley MC, Flint, Michigan; Michigan State University - Hurley Medical center, Flint, Michigan; Hurley Medical Center/Michigan State University College of Human Medicine, Flint, Michigan; Hurley Medical Center/Michigan State College of Human Medicine, Flint, Michigan; Hurley Medical Center, Flint, Michigan; Hurley Medical Center, Flint, Michigan; HURLEY MEDICAL CENTER/ MICHIGAN STATE UNIVERSITY, TACOMA, Washington

## Abstract

**Background:**

Early in the pandemic, heath care workers (HCWs) were deemed to be at high risk of acquiring SARS-CoV-2 from their patients and distanced themselves from their families. This study aimed to estimate the seropositivity of a cohort of healthcare over time while also looking for associations between seroconversion and hospital and community SARS CoV-2 exposures.

**Methods:**

This is a prospective cohort study of HCWs from patient care (PC) and non-patient care (NPC) areas conducted from April 2020 through Dec 2020 at Hurley Medical Center in Flint, Michigan (MI). The first case of SARS-CoV-2 was diagnosed in MI on 3/10/2020. In early April 2020, HCWs underwent serum testing for total SARS-CoV-2 anti-spike protein antibody and completed a questionnaire to collect data on demographics, travel, job characteristics, in and out of hospital SARS-CoV-2 exposures, and use of personal protective equipment (PPE). The serum testing and survey were offered to the same HCWs in late May 2020 and again in December 2020. Statistical analysis such as Fisher's exact test and Student's t-test were used to determine if there was an association with SARS-CoV-2 antibody status for categorical and continuous variables, respectively.

**Results:**

At baseline, 20/192 (10.4%) of HCWs were seropositive for SARS-CoV-2 total antibody with 9/20 (45%) providing PC. Job title was known for all participants however, initial survey completion was 79.6%. Eight weeks later, 13/131 (9.9%) were positive of which 5/13 (38.4%) were new seroconversions, 2/5 (40%) in PC. Eight months after the initial draw, 16/120 (13.3%) were positive with 13/16 (81.3%) new, 7/13 in PC (54%). The number of HCWs who tested positive at any time during the study was 38/192 (19.8%). No significant associations were found between seroconversion and taking care of COVID patients, any direct patient care duties, or other variables collected at the two-sided threshold of 0.05.

**Conclusion:**

No association in this small study was found between PC and SARS-CoV-2 antibody seroconversion. HCWs in NPC areas were as likely to test positive as those in PC likely reflecting community prevalence. Universal masking at the medical center and use of full PPE to care for probable and confirmed COVID patients likely prevented higher rates of PC acquisition.

**Disclosures:**

**All Authors**: No reported disclosures.

